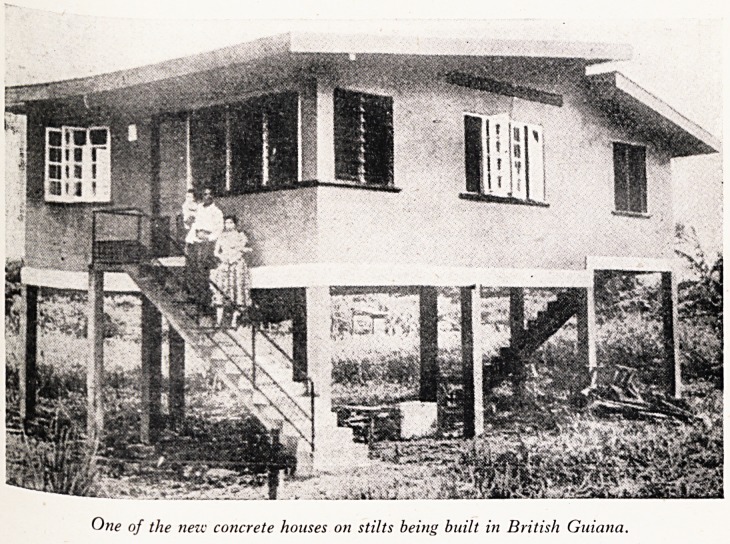# Some Notes of a Medical Visitor to the British West Indies

**Published:** 1957-04

**Authors:** A. V. Neale

**Affiliations:** Professor of Child Health, University of Bristol


### ME NOTES OF A MEDICAL VISITOR TO THE BRITISH WEST INDIES

BY

A. V. NEALE, M.D., F.R.C.P.
*Professor of Child Health, University of Bristol*

o be invited by the Colonial Office to make a tour—as Medical Visitor to British ana and the British West Indies—at rather short notice drives one quickly to s, geography and climatology, distances, air-routes and historical backgrounds, onal immunization procedures, and a rapid shaking of oneself into an appropriate dedness which, at times (as events proved), made me feel that the Commonwealth rotating round the significance of the particular place and persons with whom as at the time located. This was rammed home (and incidentally indicated that was to be no joy-ride) by the note that "at the end of his visit to each territory Visitor will discuss his observations with the Director of Medical Services and the Governor of the territory".

he terms of reference were delightfully—if a shade vaguely—stated:

He should regard all the medical services in a Colony as his proper sphere of rest: he will be able to observe conditions under which medical officers work, ange views on their problems and on recent advances in medicine; and encourage stigations of a kind that appear valuable and practicable. He will find opportunity tactfully suggesting improvements in standards of practice—but from the outset Visitor must bear in mind that he is in no sense an official inspector and this uld be clear to those whom he visits: the Visitor will consider especially doctors king in comparative isolation and, in fact, he may prefer to spend much of his with those working in outlying places. The first function calls primarily for the cise of those qualities of understanding and friendliness that mark all good titioners of medicine. The medical authorities in many colonies are deeply cerned about specific problems—including tuberculosis, child health, &c., and it he policy of the Colonial Governments to develop preventive work to the utmost, it is hoped that all participating will encourage interest and effective action in various aspects of hygiene, social and preventive medicine."

roadly, therefore, the explorations offered no limitation: it soon became evident curative medicine had a limited significance when taken out of the overall needs he community in a colony. The "State of the Public Health" (the significant title ur own ministerial Blue Books) soon demanded attention if any sort of perspective to be reached. In my journeys I frequently reminded myself of Sir J. Simon's ervations in his English Sanitary Institutions (1890, p. 298) on the inter relation- s of the personal, environmental and social circumstances, and where, in these ters, Medicine stands, and where the Government.

How far the described circumstances of our poorest labouring populations tend etter themselves, and how far they may be bettered by interference without, are stions which cannot be discussed without reference to parts of political economy which I am incompetent to speak. Indirectly, indeed, these questions are of the est sanitary importance: for the 'public health' of a country means the health of masses, and the masses will scarcely be healthy unless . . . they be at least moder- y prosperous. And although the satisfactory solutions of these questions is a task other sciences as well as the science of medicine . . . yet assuredly if that solution be given the ultimate result will be among the foremost gains which a department ublic health can have to record."

n 1st August—my day of departure—a heatwave was running in New York. My e (the wonderful B.O.A.C. Monarch) was scheduled to leave London at 8 p.m and land at New York at 6 a.m. The obvious thing was to fly in a tropical su
be comfortable on arrival. So be it. But it wasn't quite so. Owing to "adverse
we came down at Iceland at midnight: so for one hour the personal heat regu
mechanisms were needed. However, with that concluded, and back into the
plane, the interesting journey competed with the demands for sleep. At 20,0
a clear sky and moon and starlight, and a clear downward view was fortunate
masses of Southern Greenland and many enormous icebergs were seen—and
a little more sleep—we were over the wastes and lakes of North Eastern Ca
with an incredibly cold atmosphere outside and ominous snow cloud "air moun
all around and below. But we were now moving southwards and (during an ex
B.O.A.C. breakfast) the St. Lawrence came into view and then Quebec. Th
zone of Indian resistance was clearly outlined ("Three Rivers"); then Montre
so down Albany to New York (two hours late) into the heatwave coming up at
The tropical suit was the right decision!

Then southward across the sea again for 1,500 miles to the island of Puerto
which at latitude 18 was like an oven, but it was pleasant to see real tropical
tion all around and to cool off with iced coffee, whilst a short but cloud-sp
rainstorm worked itself out. By a series of plane hops across the whole of the Lee
and Windwards—including a thrilling series of alightings on land-strips with
yards to spare—Trinidad was reached in time to see some of the vast petr
plant vividly illuminated from above. Some fatigue had come over me. My oc
lobes were now saturated with the panorama of aerial pictures—and I looked fo
to a cold shower and an hour's rest in an air-conditioned room. However, it is
easy to find blessed isolation. On leaving the plane I was tapped on the shoul
a doctor whom I had known in England 15 years ago (and who had read in the
paper that I was arriving!), and so I was quickly absorbed into the Port of
medical circle for a "convivial introduction" for which I was, through the acti
the day, quite unfitted. However, this welcoming event over, I prepared for the
flight next morning. So on to British Guiana at 7 a.m. The British Viscount pla
certainly worthy of the flag. Ease and confidence simply oozes within them. Mo
coffee over Eastern Venezuela was a comfort until one fully comprehended that
was a run of 400 miles over the tropical forests, swamps and estuaries of the
Orinoco and other rivers and that they held only a great population of allig
Looking west, however, there could be seen probably the wealthiest area in the w
towards the city of Caracas—where oil is gold and the only place I know in whic
U.S.A. dollar is of no particular attraction. Our early pioneers missed it—for a
unknown except perhaps they shied at the inland mountainous contour—and i
they battled with others to acquire the most morthern of the three Guianas
although next door, unfortunately has lots of water but (up to now) no oil! The la
field at British Guiana is a fine one—a legacy of an American base in the war.
day, 12 noon (36 hours after leaving home) and I was in a new world. Heat, hum
roads and tracks with incapacitating surfaces, which are a constant menace be
the rains cause undermining and, in any case, for several miles inland and all a
the coastline of this country it is merely a low level mass of alluvial mud br
down by the rains and rivers from the higher lands stretching back right to the
zone of Brazil.

### BRITISH GUIANA

The ancient race—the Amerindians—are now relatively few in number and
been pushed right to the inner uplands where they dwell in peace and rear
(which are driven 500–700 miles to Georgetown for sale), but unfortunatel
their limited contacts with the general population (which to some 90 per c
concentrated in a 5-mile coastal strip all round the country) has allowed pulm
tuberculosis to take its heavy toll. The general population is about 450,000 an
great mixture of every shade from white to black. The dark Guianese disting himself from the intense melanosis of the descendants of the African (the story of slave transportations is everywhere evident); the small colonies of Chinese have lost none of their facial characters, although their language is clear English; there are a few Venezuelans and Brazilians; a few British "whites"—and then large numbers of true and pure bred East Indians. East Indians in the West Indies is at first confusing. Even Columbus might be, at first, a bit perplexed! However, the story of these is interesting and politically important. When the African slave trade stopped, about 100 years ago, and labour was urgently needed to develop the sugar cane and rice production, families from India were encouraged, by indenture, to come to the country. They did so in large numbers, and scattering up and down the coastline, and despite malarial devastation, increased and increased until now they are clearly numerically dominant. They are thrifty and have many subtle means of financial success. The African negro descendant is more inclined to take life easy and a good weekly dose of rum regularly absorbs his financial reserves. Hence a socio-political problem exists which, of course, the Colonial Officers are well aware. On liberation from indenture a few years ago, only a very small number of these Indians decided to accept the free passage back to India. Therefore medical matters are concerned nearly as much with the multi-racial personalities, habits and beliefs, as with the wide variety of noxious agents, indigenous to such a wet tropical land.

The Medical Services are organized on the hierarchy plan. A D.M.S. at the upper and the D.M.O. the lower end of the scale.

Minister of Housing and Health

D.M.S.

D.D.M.S.

A.D.M.S. (Curative

- Hospital

  - "Specialist" Class I Physicians

  - " Surgeons

  - " Obstetricians

  - " Ophthalmologist

  - } Medical Advisory Committee with A.D.M.S. as Chairman

  - Class II Medicine

    - Surgery

    - Obstetrics

  - Residents H.P.

    - H.S.

    - etc.

  - and Associated nursing services

A.D.M.S. (Preventive)

- M.O.H.

  - Health Education

  - B.C.G.

  - Immunisation

  - Malaria

  - Yaws

  - Filiariasis

  - Typhoid

  - School health

  - Ante-natal care

  - Post-natal care

  - and Associated nursing staff

  - Health Visitors

  - Sanitary Inspectors

D.M.O.
(Area general practice, etc.)

*Key*

D.M.S. = Director of Medical Services
S.M.O. = Senior Medical Officer
D.M.O. = District Medical Officer

There are a few private hospitals and private general practitioners in the two large towns, Georgetown and New Amsterdam. Otherwise, the territory is served by the

D.M.O.s who are under Government contract but are allowed some private prac[illegible] The rural area covered by a D.M.O. is very extensive—and the poor roads and he[illegible] rains make it no easier. It is a hard job, and some of the D.M.O.s have an Area h[illegible] pital to look after also. The rural dispensary is the G.P. consultation centre, whe[illegible] meet also the Health Visitor, District Midwife, the Sanitary Officer, and a man w[illegible] acts as Dispenser, a useful chap who does dressings, puts up bottles of medicine[illegible] acts as a minor diagnostician when the D.M.O. is off in the wilds of his round.

The roads (tracks) are rather scattered, but many follow the rivers. Houses [illegible] shacks have no names or numbers: the doctor calls in if he sees a white flag place[illegible] the door! It may be a week or more before he can be round that way again. It is [illegible] a clinician's world. Nutritional deficiency abounds. Hypoproteinosis is manifest[illegible] borderline in many, especially babies and yound children. Breast feeding may [illegible] given up in favour of cereal or flour water—strange and saddening beliefs exist [illegible] milk causes diarrhoea and intestinal parasites—thus the protein deprivation cau[illegible] generalized oedema and fatty liver enlargement or, in others, gross wasting. Cows [illegible] not very productive and the general shortage of milk is serious for a large numbe[illegible] children. Dried milks are expensive to import and Government subsidy is uncert[illegible]

Infections are common at all ages, enteric fever, ankylostomiasis, amoebic [illegible] bacillary dysentery. Pulmonary tuberculosis is rampant in some areas; leprosy [illegible] sporadic, but the doctors are alert to the early diagnosis of the cutaneous and neur[illegible] lesions, thus quick chemotherapy can cure it and attempts are being made to cl[illegible] down gradually the rather large leprosarium—but there is the ever-menacing r[illegible] of more Hansen's infection moving in at the Brazilian frontier where many more c[illegible] exist. The D.M.O.s do all they can to co-operate and help in the health educat[illegible] programmes in the villages, making full use of the Community Hall which is s[illegible] (usually next to the Police Station!) in each area. A feature of British Guiana is [illegible] large sugar plantations, all very well organized under the large Sugar Corporati[illegible] and have their own whole-time doctors in their Medical Services. These are g[illegible] jobs but so much trouble arises from the excess "compensationitis" which abou[illegible] in the labourers. The hot climate does not, at the best of times, lend itself to [illegible] energetic approach, and minimal injuries are made the most of. Litigation is comm[illegible] even over trifling matters, and seems to be, in part, an outlet for emotional unres[illegible] for a passing phase of self-importance. A very minor legal action in the courts [illegible] attract an endless following as witnesses and observers—almost a childlike gather[illegible]

Georgetown is in part quite a modern city—at least that part which was repla[illegible] after an extensive (and useful) fire a few years ago. Bookers, the great commer[illegible] firm, have developed the place well and there is an excellent natural history muse[illegible] and zoo, and a fine general library in the city. The lake in the zoo contains an ass[illegible] ment of alligators which soon make themselves apparent if a bird alights on [illegible] water. A walk round the town is very pleasant—good houses, many built up on stilts [illegible] counter the floods (see Plate II). The Anglican Cathedral is an enormous and i[illegible] pressive all-white wooden building. There is a canal system of flood drainage do[illegible] the centre of the main roads and one has to be careful after dark. These arrangemen[illegible] together with the uncertain insulation of the common domestic surface latrines, te[illegible] to risks of typhoid, Weil's disease and ankylostoma duodenale. The Sanitary Inspec[illegible] is a very important man indeed, and the D.M.S. sees to it that they are never in sh[illegible] supply.

The country has many rivers, and alligators are very numerous but only occasio[illegible] ally dangerous. Snakes are more troublesome and the common scorpion is a shocki[illegible] nuisance from its attacks.

The extensive ricefields, worked on almost entirely by the East Indians, provid[illegible] the staple diet for many. But only those capable of producing first-class crops a[illegible] allowed to grow rice. The Government is very reluctant to have to import rice—it [illegible] too great a drain on the national budget and exchange.

The main hospital—"The Public Hospital"—is a fantastically busy place. The[illegible]

PLATE II

*One of the new concrete houses on stilts being built in British Guiana.*

is a perpetual queue of people waiting to be seen—many hoping to get a mysterious bottle of a placebo—but the clinical material in the wards is wonderful. Disease is rampant and advanced anaemia and infection due to hookworm is treated at out-patients if the Hb. is 30 per cent. and admitted if below 20 per cent. In the infectious diseases wards—wards in the main hospital campus and only slightly demarcated from the others—I saw 40 enteric fever cases (all ages) with nearly the whole range of symptoms and signs but, owing to chloramphenicol, hardly any complications. Many of the population must be (by virtue of subclinical infections) immune to the typhoid fevers, but even so no organized scheme for preventive typhoid vaccine has yet been involved. Serious grade rheumatic heart disease, syphilitic aortitis with aneurysm, liver cirrhosis with associated neuropathy and "flapping" tremor, severe respiratory infections, hepatitis, Weil's disease, amoebic liver abscess, were all about, and hypertensive disease and cardiac failure in the Negro groups and chronic emphysema in the East Indians (and rarely the other way round) was of interest in terms of racial susceptibility. Filariasis could be seen in its acute lymphangitis form and many cases of ultimate limb elephantiasis were seen in the town. Tetanus is common in both babies and adults and the mortality is fairly high. The Surgeon-specialist (in fact a very general surgeon) has almost infinite scope in visceral and limb surgery. Thoracic Surgery and Neurosurgery are hardly existent yet, but are noted as a need. Ocular disease is mainly cataract and glaucoma, but "nutritional corneal ulcer" is frequent in children.

Preventive medicine is given a high priority in all the schemes. The D.M.S. has his "Health" section which acts, so to speak, nationally. Doctors attached to the health department tour the land with Health Visitors. B.C.G. vaccine and anti-filarial schemes are active. Malaria, until recent times almost a hopeless problem in a rain and *Anopheles*-ridden country, is under firm control, thanks to Dr. Giglioli and his D.D.T. mobile teams. The hospitals are very overcrowded. I have seen three infants in one cot. Home Care and some Domiciliary Nursing Services are needed. Hitherto, the housing has been very bad (there are still some of the original barrack slums of the slave days), but that is improving rapidly. The wooden shacks are being replaced by concrete structures (see Plate II). The people are being interested in "self-help" housing and building arrangements and many can be seen building their own homes, under supervision. Town planning is good and an orderly scheme is now proceeding, which is in contrast to the previous shanty-villages.

The Education Officer has a permanent headache trying to keep up with the increasing population: ordinary schools can barely meet it, but there is an excellent group of Higher Schools for those who can get in. A School Health Service is being developed. Many schoolchildren have moderate protein and vitamin deficiencies and the hookworm is a great nuisance in this age group; and occasionally sickle-cell anaemia with splenomegaly is detected.

The fecundity in the coloured population is high, but there is a good deal of pelvic sepsis, luetic disease and gonococcal infection. Gynaecological disease is common and there is a very high incidence of ectopic gestation. There is a high incidence of pregnancy toxaemia, and eclampsia is almost a daily occurrence. The Medical Staff live in quite good houses in the general campus of the Public Hospital and excellent clinical meetings are held each week which is a very creditable performance and a worthy method of neutralizing the otherwise possible apathetic effects of clinical isolation and the almost insufferable burden of feeling so very remote from any centre. They are certainly delighted to have a medical visitor and my time in British Guiana was a valuable experience—both ways—I ventured to hope.

### TRINIDAD

Distances are elusive in the Caribbean. It is over a thousand miles from Trinidad to Jamaica: so it is not surprising that there is very considerable autonomy in the various parts of the B.W.I., each island is truly insular and keenly aware of its own historical background. Trinidad (Columbus's name for the island after seeing t three mountains) carries some residual indications of the original Spanish occupatio but these are nearly smothered now by the general modernizing, especially arou Port of Spain, its largest town, and the financial success of oil production in t southern half of the country. It is the only island in B.W.I. which has any reasonab hope, in any year, of balancing its budgets. The political situation is very compl As in British Guiana, there is a rising percentage of the descendants of East India (but they all resolutely regard themselves now as West Indians) out of a total 700,000 people. The country is attractive, flat lands to the west and mountain fore to the east. The sunrise and the sunset is nearly always a wonderful picture, and t length of daylight varies little more than half an hour all the year round. There unlimited interest in this country. The forests harbour large numbers of monkey one variety being a carrier of the yellow fever virus. Snakes also exist, but their m nuisance value is their liking for the citrus trees which fill the little valleys and t banana trees—thus the fruit pickers need keen eyes.

Research on viruses has reached an advanced stage; there is an excellent institu (financed by the Rockefeller Foundation) in which Dr. Downes has tracked down wide variety of specific viruses infecting animals and man. Public health is excellent organised in Trinidad—in fact, there is a tradition in that line, but there is a neve failing need for alertness in this efficient hygienic control. Infections would otherw be utterly rampant. Even as it is, the picture of England in 1900 is still to be seen diphtheria, typhoid fever, tetanus, and puerperal sepsis, with malaria, dengue, yello fever here and there. Rheumatic carditis, with an exceptional incidence of aort valve damage, is rather common in children and in adolescents, and syphilitis aorti is a troublesome problem in the middle age groups.

The administration is similar to British Guiana, but the Colonial Hospital at Po of Spain is an attractive place and the clinical material abounding. A Registrar wou have a wonderful experience and I hope that some such visiting arrangement mig be developed. Every conceivable variety of disease and pathology is there. There a some specially troublesome matters, such as severe waves of gastro-enteritis as additional factor, and often a lethal addition, to the common protein-deficien syndromes: and whole sections of the obstetric department given to the care of seve toxaemia of pregnancy—and yet, as throughout most of the B.W.I., there is rar a case of pulmonary embolism. It may be something to do with the local diets a such matters as blood lipoproteins and fibrinogen—but all this calls for medic research on a big scale, and Trinidad would be a good place to do it because the vario races, whilst forming cohesive communities, have preserved their racial ways of l and their special food habits.

Clinical pathology and bacteriology is really exciting: the distribution of adnorm haemoglobins and the sickle-cell anaemias; the bacterial and the amoebic intesti infections; the several spirochaetal diseases, including yaws and syphilis. Tuberculo is dealt with most efficiently—the Caura Hospital is a model of what can be done effective combination of chemotherapy and thoracic surgery, and a follow-up in detail including a rehabilitation-occupational centre. Tuberculosis has carried strong anti-social stigma, thus these good preventive and curative services are righ praised. Leprosy cases are still incarcerated in a small island three miles offshore an enlightened medical officer liberated a few cured cases a year or so ago, but th were quickly chased up and sent back by the indignant and fearful administrati authority! I consider that this is a sad affair and needs a real fact-finding inqui However, as in British Guiana, Hansen's disease is now being recognized early (n cases are quite rare) and, so we may confidently hope, in a quickly curable stage.

Trinidad is full of interest to those that seek around: the Golden Grove Pris for Adolescents has excellent humanizing influences for the local equivalent of t Teddy Boys; and an Approved School for boys of 8–12 years is run on simple fai and common sense; in a rural zone there is the (now famous) Imperial College

Tropical Agriculture, where students from every country in the whole Commonwealth get together and where agricultural research is having a great influence on the economic productivity in all tropical and sub-tropical countries. An area of Port of Spain—"Shanty Town", to me the world's worst collection of derelict but inhabited shacks—is such an eyesore that the local authority built tall and colourful advertisement signs to obliterate the site during a recent Royal visit to the island! A day with a D.M.O. was interesting—all morning as a "state doctor" at a "Health" Clinic, which is really a long line of people seeking the bottled cure for every symptom (neurosis is common to the East Indian and to the Negro) or the essential vermifuge. An afternoon doing his private practice, on a ready-cash system of 2 B.W.I. dollars minimum, and visiting his local cottage hospital. The job is good, full of interest since the patients' personalities are each so unique and many dwell in the mystic; but the D.M.O's. clinical sense must be clear—in his hospital he deals with a good range; diabetes mellitus, toxaemia of pregnancy, an enteric case (there are no isolation hospitals and "fevers" are just general medicine), tropical ulcer, chronic emphysema with failing right heart, and so on. I was glad to see the Princess Elizabeth Home (our present Queen opened it) for physically handicapped children; to Dr. Reed, the local visiting G.P., it was clearly the apple of his eye and a treasured hobby of his life. The surgeons in the Caribbean hospitals spend a whole day in rather small operating theatres working through long lists; air-conditioning is partly successful, but the surgeon's comfort is not guaranteed on some hot humid days. The obstetricians are a heroic lot, there is hardly a moment's peace. The Trinidadians, like most West Indians, combine the social with a high biological potency, thus reproduction has a raised frequency and the midwifery services, hospital or domiciliary, are never slack. The mechanisms of birth rarely cause trouble, but the medical aspects—toxaemia, anaemia, nutritional disorders, pyelonephritis and gonococci—do.

Thirty miles south the other sizeable town—San Fernando—is a mixture of the old Spanish influence and the modern oil world. A new hospital, 300 beds, a keen group of doctors and a first-class nursing school staff, attracted me, and I was soon "at home" when I found that the Matron was a Sister on one of my wards in Birmingham 12 years ago, that the Assistant Matron (Miss Waterman) was at the B.R.I. until two years ago, that Dr. Wattley (Physician) is an M.D. Bristol (was Dr. Orr-Ewing's House Physician), that Mr. Briggs, Obstetrician, knew Bristol in some detail, and that Dr. Bruce Symonds, who had been a guest at our South West Paediatric Club meeting at Bristol, had a main hand in the Child Health Services in Trinidad, and is specially interested in the "Sugar Babies". I required no further introduction. A nice climate, sea-bathing nearby, a visit to the newer oil drilling grounds, and the magnificent medical buildings and oil research station of the Petroleum Company and a walk over the famous pitch-lake, all toned in well with a surfeit of daily clinical activity. Strange forms of cardiomegaly in childhood were mingled with distressing cases of tetanus neonatorum admitted from the country districts. A cyanosed black (sic) woman, aged 36, arrived and died in severe dyspnoea—necropsy showed the Tetralogy of Fallot. I relieved this day by a visit to a D.M.O. who took me over a sugar factory, the crushing machiner is immense and heavy and serious accidents occur.

The surrounding country of Siparia, contains every evidence of a struggle between human life and the economic limits of false standards in a country spuriously rich in oil but truly dependent on agriculture. A man is quite content to live with his large family in a two-roomed pile-supported shack but keep a car in the space underneath—petrol is very cheap and plentiful. There is no Children Act: deprived infants are numerous. "Baby farms" are in evidence. I visited one with 15 children under 2 years, all in a three-small-roomed wooden house—under the care of a woman receiving an occasional dollar from a baby's mother. The arrangement, of course, is amazingly loose, but typical of a country struggling to find some order out of the chaos of excess population, a matriarchal system of life and a direct effect of the sudden granting of "freedom" to the negro slaves a hundred years ago. It has never really sorted itself out—and all this is now of some significance in the troubles the proposed B.W.I. Federation and future Dominion status. The medical aspe are complex enough, but the socio-political position is only stable through a contin ostrich-like view of a very limiting commercial strength. But Trinidad is facing problems at last (at least, so says their brand new Government) and I shall certa follow Caribbean affairs with great interest and some local knowledge.

The Windward Islands, comprising St. Vincent, Dominica, Grenada and Lucia, provide a wonderful tour over a distance of about six hundred miles. Ea island is so different and yet each is essentially land around an ocean mountain.

### ST. VINCENT

One hundred and fifty square miles, population 74,000, and famous for its arrowr (it provides 70 per cent. of the world's supply), is a well-organised island with go medical services and friendliness. Kingstown (18,000) has the central hospital wh is a meeting-place for all the doctors and Mr. Gun Munro, the Surgeon, is a fir class leader. We had several rounds and here I saw an incredibly large left hyd nephrosis. The D.M.O.s and the domiciliary midwives have to travel up and do and across the island—the coast road is fairly good, but the rest are tracks only. O D.M.O. moved around on horseback, his journeys running through banana a coconut plantations on the leeward side and through the arrowroot fields on windward. The only car possessing a reasonable life is a Land Rover: and petro 75 cents a gallon. The population is almost entirely negro with its easy-going li Each island has its own government and system of ministers who, through t Administrator, is responsible to H.M. Governor who resides in Grenada. It is a perpetual struggle between appointed power but without available money to ma any visible progress. The Nutritionist is as important as anyone—it needs a lot advice to get the most out of the limited home produce (again milk is scarce) a wean mothers off their use of "bush tea" or an undue faith in a bottle (usually a desirably blue) of coloured water hanging over the house door! "Bush tea" can unsafe—digitalis or aconite poisoning cropping up occasionally.

On this island I met Dr. Lamb (Lecturer in Pathology in Birmingham when was a student, 1923): he is now ordained in the Anglican Church but places special experience in forensic pathology at the full disposal of the judicature of t Windwards—and the judges told me of his value in sorting out the subtle tricks homicide in these remote parts. There are many thinking people on the island, fac up to the legacies of an isolation for many years. There is a general happiness— sun, the air, and the sea, lend themselves to that, but there has been barely any attem at any inter-island co-operation so far. Each has been content and satisfied to exp its bananas, or its grapefruit, sugar, coconuts and copra, cocoa beans or nutme and import its white flour, tinned foods and English potatoes. Hence, boats come a go in the pretty harbours, yet only a handful of St. Vincentians may know anythi about the Grenadians only 60 miles away. Clearly, one of the first things in Federati is to get better links and the medical staffs on the islands must learn about wi co-operation and interchange of knowledge and ideas. W.H.O. has given aid in t way by their experts conducting overall attacks on yaws—which is easy to clear ev with one massive dose of penicillin, and syphilis—which is difficult to clear becau the re-infection rate is so high. Tuberculosis in these Windward Islands needs inte island team work; the present arrangements are quite unsatisfactory.

### DOMINICA

Area, 300 sq. miles, population 60,000, and named by Columbus on his third vi to these seas, has many signs of its long French occupation and the terrible Franc British battles on its shores, until the French decided to move out and live in pea on their own West Indian islands—Guadeloupe and Martinique—which can be se about 50 miles away. The general plan of medical work is similar. The only English church has a congregation of about 100—the rest of the people are Roman Catholics. The colourful pageants and Saints' Days arouse the people periodically from the lethargic influences of the brilliant sun and heat of the day and put them into a happy mood for the long moonlight nights, when the air is rent by drones of a "Steel Band" (empty oil drums of various sizes). Puberty and fecundity seem sometimes to synchronize. The long warm moonlight nights in these tropical latitudes carry their own social problems. An ante-natal clinic in Dominica shows at once some reflections of this—95 per cent. are unmarried—the matriarchial system is accepted and the man's place in nature is to be effectively biological. There is a new and well-organized hospital with very good facilities, colourful wards, and each patient has a bedside dressing table! A round revealed a good selection—appendix abscesses, the usual "kwashiorkor"-like syndromes, several ununited fractures (a troublesome thing in the B.W.I.), "fevers" of all varieties, and the ever-troublesome tuberculosis. A R.C. Sister of Mercy is an acting Hon. Surgeon (M.D. Louvain) and her skill is admirable.

This is the only island wherein reside the few (about 400) remaining Caribs who originally moved from the Central American mainland. During my stay on this island, several tons of U.N.I.C.E.F. dried milk arrived for the young pregnant women and nursing mothers and the young children—enough, we considered, for about three years. Breast feeding continues for about nine months (the R.C. welfare centres have a hand in this) and maternal care is good. Birth has its risks, however, even there: I was asked to see a typical case of quadriplegic cerebral palsy in a child following a difficult breech delivery and severe clinical anoxia.

The island prison contained 68 men and 1 female! The Mental Hospital was next door and the prison governor supervised it—the incarceration was not very dissimilar. However, to the comfort of many, there is a magnificent dental treatment centre in Roseau, which the Senior Medical Officer considers is a mainstay to the peaceful population. Each of the Caribbean Territories prides itself on its own rum. Dominica converts all its sugar cane into rum and even imports more from the nearby French islands, reaching a total annual island turnover of 120,000 gallons in home consumption. This might have something to do with the troublesome incidence of tuberculosis; however, cirrhosis of the liver is rare and alcoholic poisoning or neuropathy is practically unknown. Perhaps the limited protein but high carbohydrate and fruit diet is helpful after all. As Roseau jetty takes in the rum, the other small port, La Souffrier, exports large amounts of fresh lime juice.

There was a sad moment just before stepping into the boat to get to the seaplane. A friend of the S.M.O. who came with the party to see me off asked me to look at his swollen tongue. I did, there was a massive hopeless epithelioma; with some quickly gathered words I departed—sorry for him and for myself in having to leave so soon—but happy to know that the island volcano was still quiet, although an outburst with a wide river of lava, as in 1930, might again occur at any time.

### GRENADA

Area, 130 sq. miles, population 81,000, Cap St. Georges 21,000. The Governor of the Windwards and the Chief Justice live on this island. It is a lovely air-trip from Trinidad, the coral seas and the blue Caribbean being seen at their best. But Grenada was seen at its worst. Hurricane Janet had recently devastated the island. Great plantations were terribly ripped up, so that the commercially precious and almost unique crops of nutmeg (this island supplies 70 per cent. of the world's nutmegs, sold principally to U.S.A. at a good dollar exchange); and the heavy crops of cocoa beans, were nearly ruined. Houses literally disappeared into fragments. I saw a brave population rising to this emergency, and every available space is now planted with a quick-growing biannual-cropping banana tree; it will take seven years to recultivate the destroyed nutmeg and cocoa trees.

All this added to the concern of the S.M.O.: the sanitation and housing provided a major crisis, and it emphasized that the Windwards are only a geographical groupi —historically and economically they are each distinct. In fact, Grenada (which h a broadcasting centre) sent sympathetic messages to St. Vincent that the hurrica was coming her way—but the hurricane unexpectedly changed its course and rushe over Grenada instead. The Colonial Development and Welfare Funds of the Coloni Office were immediately called upon—and help was given. There is thus a very sl recovery after a few hours of raging wind moving in a circle at 120 miles an hour a hitting everything first left to right and then right to left—and it is the only time th heavy rain is nearly horizontal at land level—all other downpours, however hea are nearly vertical and even windows need not be closed.

There are 20,000 children of school age, and increasing at about 2 per cen p.a.; many schools were wrecked in the hurricane—my sympathies were with the Educati Officer who seemed to have a nearly impossible job. As in St. Vincent and in S Lucia, there is an excellent group of higher schools where good general, gramm and technical and science courses are given.

At the Colony Hospital the old classical genes were seen in clinical effect—muscul dystrophy, fragilitas ossium, microcephaly—and a rather rare disease in the Carib bean, athetotic form of cerebral palsy due to neonatal kernicterus. It is noteable th less than 1 per cent. of the women in B.W.I. are Rhesus negative; hence haemoly disease with severe jaundice is a clinical rarity. The wards were a veritable museu of material; lymphogranuloma inguinale, severe rheumatic pancarditis, infecti endocarditis, "infectious jaundice", various treponematoses—and the surgeon show me his skill in dealing with several amoebic liver abscesses and some of the ol fashioned massive ovarian cysts and uterine fibroids. The clinical laboratories we very modern with excellent up-to-date equipment—large binocular microscop special centrifuges, and all sorts of things which I did not expect to find in a sm Caribbean island, and research was in progress on intestinal infections and an biotic action.

The Governor of the Windwards discussed with me a wide range of things tuberculosis, nutrition, thoracic surgery, island economics, family planning as urgent need, the hope (as remote as it may seem) that there may be a balance betw numbers and agricultural possibilities. Just to add to the troubles, a rat probl has existed since the hurricane—the rodents attacking the sugar cane and swe potato crops, and causing also small epidemics of Weil's disease. Although surround by the sea (and many flying fish) fishing is poorly organized and supplies very u certain and irregular and there are no means of refrigeration, so in a glut many go waste. The "health education" schemes—and many good thinking people give ti and energy to it—sometimes come to nothing when it is discovered that the pare are selling their fresh hen eggs only to buy white flour for their infants. The dri milk scheme also had its problems, many (and even some of the Health Visitors a midwives) believing that the partly defatted dried milk was no good—that the gift w a fraud—so we spent a lot of time talking about the essential and important protei As I left I saw a ship depart after loading 8,000 branches of very green bananas a I was nearly caught in the group of "film stars" who were gathering on Grenada f the making of the new film *An Island in the Sun.*

### ST. LUCIA

(Pronounced "Loosha".) Area 253 sq. miles, population 56,000. Capital, Castri 24,000. Even Americans call to see this lovely island in their Christmas-time Carib bean tours. There was a useful massive fire in 1951—so that many tidy and attracti buildings now exist in Castries beside a perfect harbour, which in days past acted a coaling station and earned for the shipping master in London some £30,000 a yea although he never saw the place: the whole thing ceased after a visiting commissi became aware that the coal was carried by women, on their heads and stripped to th waist!

The common language is a mixture of French and English. This was the island which the French would not let go until Napoleon implied he "couldn't care less" for the W.I.—so the military men got out, but many French civilians remained. There are some good four-storey flats and excellent amenities, including good hotels, in Castries. There is a Medical Centre from which the S.M.O. directs and organizes the island's Medical Services. The Centre contains general medical clinics, a Child Health Clinic (the only place in the B.W.I. where I saw the term used) and maternity clinics. D.M.O.s worked in this Centre and worked out in the rural zones at set times. A general hospital was nearby, so everything seemed geographically convenient, and all the doctors seemed to be gathered in a happy social group. In these island services much depends upon the personality of the D.M.S. or the S.M.O. In St. Lucia this was well supported by the Specialist-Surgeon, who radiated confidence, order and versatility. We all gathered at a clinical meeting to see and discuss—interlobar empyema, osteomyelitis of the pelvis, osteomyelitis complicating fractures, staphylococcal abscess of the abdominal wall, typhoid bone abscess, severe typhoid in "coma vigil", insulin-resistant diabetes mellitus (a peculiar type seen in the W.I.), acute facial yaws, congenital hydrocephalus, congenital syphilitic osteitis with good old-fashioned sabre-tibia—and finally a straightforward patent ductus arteriosus. I only wished I had a group of students with me. We suspected a bizarre neuritic syndrome as possible leprosy, and a report came in that a few cases of Bilharzia were present at the neighbouring town of Souffries—but, at the same time, the ever-faithful sanitary teams had cleared up the mosquito breeding grounds and no malaria had been seen for months. The clinical interests need never lag in St. Lucia. On the last day I arrived back in Castries after a day with a D.M.O. in the country, seeing hundreds of patients trying to make themselves understood to us with the strange Franco-English patois. The day was long, but we saw the direct remains of old slave camps away in the hill jungle and as remote as man may be. They kindled some sad memories of our bygone colonial mistakes.

### BARBADOS

Area 166 sq. miles, population 223,000. Capital Bridgetown. When the English landed in 1627 and took over this nearly flat coral island (which is about the size of the Isle of Wight), it was quite uninhabited, although there were indications of a remote occupation by Caribs and later Arawaks from South America. The island story is a classic—whites, slaves and sugar cane. Now, there are only about 7,000 whites—the rest are all of African negro descent, and the island is supersaturated with human life. Every available square yard, as is well seen from the air, is used for cane or sweet potato, and houses are squeezed into the odd patches and around the coast: the cows feed on the narrow grass verges of the roads. Some houses are built with coral blocks and remain beautifully white. Barbados is a busy place—they have called it "Little England"—with administration, including the Parliament and the Ministers, in a sort of Lilliputian State. Difficulties abound—for instance the 30 lepers at Lazaretta cost £300 per person p.a., all burnt-out cases occupying many acres of valuable land and buildings. The persistent prejudice about leprosy detracts from the now much more important epidemiology of tuberculosis.

Private general practitioners do most of the medical work in Barbados. The pattern is distinct from the other islands. There are a few D.M.O.s who each act also as an Area M.O.H. Co-operation is not good, mainly perhaps because, even now, the island is divided up into Churchwarden Areas, the original governing power of the slave population being through Churchwardens! The Anglican Church is strong, but now there are thirty different "churches and chapels"; and, in any case, the "Churchwarden" is now a politician. The slave owners, no doubt, originally gained some release of conscience through the Churchwarden system! Medical and social problems are common and not helped by the regular increase of the population by 2,000

p.a. There is an illegitimacy rate of 57 per cent. (1950) and 61 per cent. (1955), an infant mortality rate of 134 (1955). There is the usual infinite range of diseas all ages—a medical or surgical registrar would be able almost to “complete his e cation” in Barbados—there is, in fact, literally too much of it; and every woman o 30 years of age has palpable fibroids! One hears little about tonsils, but there interminable lists of hysterectomies.

Everybody in authority seems to be planning *something* in Barbados, but essentials of “Family Palnning” are only just existent. If it doesn't become effec soon everthing will crumble. The D.M.O. and the hospital staffs are usually o whelmed with pressure of cases, mainly because no serious attempts to push he education or home-care have been taken until quite recently. A year in Barbado certain to give any doctor or nurse or social worker a completely new approach life, and certainly may have happy memories of bathing in the clear coral sea; watching the subtle skills of the Barbadian with a cricket bat and ball—some which we hope to see in England this summer of 1957.

Dr. William Hillary (1697–1763), physician in Barbados in 1746, wrote on “Obs vations on the changes of the air, and the concomitant epidemiological diseases, the island of Barbados” and included an original and most accurate description tropical sprue, but at the same time he noted the island to be pleasant and m preferable to Jamaica.

As a young doctor (30 years ago) I would have revelled in the clinical world the B.W.I. It may be summed up: “Many of us in Britain are apt to forget the e mous sea distances between the countries and islands which make up the Bri West Indies. They form a vast semicircle, which covers over 2,000 miles, start on the Central American mainland in British Honduras, and passing through Cayman Isles, Jamaica, the many Leeward and Windward Isles, Barbados, Trinidad, to end at British Guiana on the northern coast of South America. forget that the journey from Jamaica to Trinidad is roughly the same distance that from London to Moscow or Istanbul. Yet these scattered lands are closely lin racially; less than 5 per cent. of their peoples are of unmixed European descent, the majority are African Negroes, with a proportion of East Indians and a few Chin They are also linked economically through their agriculture; all of them prima grow sugar or bananas, with smaller quantities of citrus fruits (Trinidad stands al in having a rich supply of oil and asphalt). A tropical climate modified by the close of the sea is common to them all; and they share too the same historical developme It is hardly surprising, therefore, that they have the same medical problems.

“Anyone from Britain visiting a general hospital in the West Indies would struck by the amount and variety of the diseases that can be studied there; but ne always there are the dreadful complications of poverty and malnutrition to mo the clinical picture and hamper treatment. Yet, superficially, the similarities British medicine are perhaps more striking than the differences. Typhoid fe malaria, sickle-cell anaemia, lymphogranuloma venereum, and some specific eff of malnutrition (especially in children) would be the only unfamiliar diseases the British doctor would commonly see in the general hospital. And he would n with interest the scarcity of emphysema, mitral heart-disease, and neurological con tions such as disseminated sclerosis, subacute combined degeneration, and ta (despite the prevalence of syphilis). On the other hand, acute lobar pneumon paraplegia (often due to cervical spondylosis), acute rheumatic fever (in contrast the rarity of mitral stenosis), hypertension and its complications, and (possi linked with hypertension) eclampsia are common.”

Strangely enough, cigarette smoking is common, but I heard very little ab carcinoma of the lung.

The Teaching Hospital and Medical School of the University College of the W Indies, at Jamaica, is flourishing. I agree with Dr. Ian Grant (who recently pai goodwill visit to the Caribbean branches of the B.M.A.) that, with a continuance goodwill on both sides, a very real improvement will take place, both in the Government-doctor relationship and in a much more efficient medical service for the people.

After these eight weeks of action—10,000 miles by air—the plane moved off the runway at Barbados at mid-day on Saturday, 30th September and I was home in Bristol on Sunday evening—this sudden jolt from one world back into another was perhaps the greatest maladjusting disturbance of the whole affair—a more sensible person would have taken a ship and more gradually eased the mind.

Since that is now all over I am finally reminded by the Colonial Office that:

"It will contribute considerably to the success of the scheme if the visitor is prepared to devote a fair amount of time and thought to the Colonies he has visited, when he returns home. If he has succeeded in arousing interest or in getting some project or other going, he may expect to receive quite a flow of correspondence in that connection which will undoubtedly make demands on his time."

Exactly! I have certainly accepted a long-term responsibility. If any young doctor wants guidance about work in the British West Indies, I am at his service.

## Figures and Tables

**Figure f1:**